# Supplementary material for: Scaling up the primary health integrated care project for chronic conditions in Kenya: study protocol for an implementation research project
Source: BMJ Open. 2022 Mar 16;12(3):e056261. doi: 10.1136/bmjopen-2021-056261 (PMC8928278; doi:10.1136/bmjopen-2021-056261)
Supplement: Supplementary data [file bmjopen-2021-056261supp001.pdf]

Scaling up the Primary Health Integrated Care Project for Chronic Conditions in Kenya: Study Protocol for an implementation research project

Appendix

Table A.1 Key features of Busia and Trans Nzoia counties (2018)

Characteristics	Busia county	Trans Nzoia county
**Population**	893,681*	990,341*
**% population aged 15+ years**	52%	53%
**% females**	52%	51%
**Live expectancy at birth**	47 years	60.5 years
**Poverty level**	64%	50%
**Estimated hypertension prevalence**	22%	27%
**Estimated diabetes prevalence**	1.5%	2%
**NHIF coverage**	31%	20%
**Number of public health facilities**	184 community units served by 47 dispensaries, 12 health centers, 5 sub-county and 1 county hospital	198 community units served by 38 dispensaries, 8 health centers, 6 sub-county health facilities and 1 county hospital
**Number of patients with selected NCDs serviced in AMPATH sites**	6060 patients with hypertension, 1113 with diabetes and 200 with cervical cancer.	4,375 patients with hypertension, and 2,089 with diabetes.

Sources: PIC4C project protocol (2018, unpublished); * Kenya National Bureau of Statistics (2019). Kenya Population and Housing Census. https://kenya.opendataforafrica.org/msdpnbc/2019-kenya-population-and-housing-census-population-by-county-and-sub-county?county=1003270-busia (last accessed 20 March 2021).
